# The interrelationships among insomnia, frailty, anxiety, and cognitive function in middle-aged and older adults: a cross-sectional study

**DOI:** 10.3389/fpsyt.2026.1783363

**Published:** 2026-06-12

**Authors:** Xue-Qiao Wang, Yan-Xin Wang, Zhao-Hao Ye, Zhi-Lin Zhang, Xun Pan, Xi-Chen Wang

**Affiliations:** Department of Neurology, The First Affiliated Hospital of Anhui University of Chinese Medicine, Hefei, China

**Keywords:** anxiety, cognitive function, frailty, insomnia, moderated mediation

## Abstract

**Objective:**

This study examined the complex relationships among insomnia, anxiety, frailty, and cognitive function in middle-aged and older adults, testing whether frailty moderates the mediating role of anxiety between insomnia and cognition.

**Methods:**

This cross-sectional study included 108 insomnia patients recruited from community and hospital settings between August 2025 and February 2026. Assessments included the Pittsburgh Sleep Quality Index, FRAIL Scale, Montreal Cognitive Assessment-Basic, and Hamilton Anxiety Rating Scales. Moderated mediation analysis was conducted using the PROCESS macro (Model 7).

**Results:**

Pre-frailty significantly moderated the insomnia–anxiety relationship (PSQI × Pre-frailty: β = 1.029, p = 0.017). The indirect effect of insomnia on cognition through anxiety was significant in both robust (β = −0.053, 95% CI [−0.111, −0.004]) and pre-frail individuals (β = −0.090, 95% CI [−0.179, −0.007]), with a significant index of moderated mediation (Index = −0.037, 95% CI [−0.101, −0.0003]), indicating that the anxiety-mediated pathway was stronger in pre-frail individuals. Both frailty (β = −1.3, p < 0.001) and insomnia severity (β = −0.176, p < 0.001) independently predicted cognitive function.

**Conclusion:**

The anxiety-mediated pathway between insomnia and cognitive impairment is active in both robust and pre-frail individuals but is significantly amplified in the pre-frail state, identifying pre-frailty as a distinct psychologically vulnerable window. Integrated interventions targeting insomnia, anxiety, and pre-frailty may be particularly beneficial for preserving cognitive health in middle-aged and older adults.

## 1 Introduction

Insomnia, characterized by self-reported difficulties with sleep initiation and maintenance (1), is a prevalent condition that significantly impairs quality of life, particularly among middle-aged and older adults (2). A robust body of evidence links insomnia to cognitive decline, with studies demonstrating that greater severity and longer duration of insomnia are associated with an increased cognitive burden (3, 4). Notably, subjective poor sleep quality in older adults with insomnia correlates with deficits in attention and overall cognitive performance (5), whereas interventions such as cognitive behavioral therapy for insomnia (CBT-I) can improve cognitive outcomes (6). However, the intrinsic mechanisms underlying insomnia-related cognitive impairment remain incompletely elucidated.

In this context, research targeting middle-aged and older adults should not only focus on disease management and the delay of disability, but also on the fundamental concept of “healthy aging, ” with the aim of maintaining and improving positive outcomes such as quality of life, functional independence, and social participation (7). Older adults consistently rank cognitive health, emotional well-being, and the ability to remain active and autonomous as central components of their quality of life (8). Sleep quality, in particular, is a critical yet often overlooked determinant of these outcomes—poor sleep not only impairs daily cognitive performance but also undermines the emotional resilience and physical vitality necessary for sustained social engagement and independent living (9). Understanding the mechanisms through which insomnia affects cognition, and identifying factors that may exacerbate or buffer this relationship, is therefore essential for promoting healthy aging and preserving quality of life in this population. The present study addresses this need by examining the interrelationships among insomnia, anxiety, frailty, and cognitive function within a moderated mediation framework.

However, the inconsistent findings regarding the anxiety-cognition relationship suggest the presence of a moderator. Clinical and epidemiological studies consistently report a high comorbidity between chronic insomnia and anxiety symptoms (10, 11). Contributing factors may include the long-term use of hypnotic medications and significant psychological burden (12). For instance, a cross-sectional study found that approximately 47.9% of individuals with insomnia exhibit significant anxiety symptoms (13), while a systematic review estimated the prevalence of insomnia symptoms in adults with depression at 78% (14). Furthermore, both insomnia and anxiety symptoms are independently associated with cognitive impairments (15, 16). Nevertheless, the relationship between anxiety symptoms and cognition is not deterministic, as evidenced by inconsistent findings across diverse clinical populations (17–19). This heterogeneity suggests that the negative cognitive impact of anxiety may be contingent upon other moderating factors.

Frailty, a clinical syndrome reflecting diminished physiological reserve and increased vulnerability to stressors, may serve as a critical moderator explaining this inconsistency. Frailty is not only an independent risk factor for cognitive decline (20) but is also associated with elevated levels of anxiety and depression (21). We hypothesize that frailty constitutes a state of physiological susceptibility that amplifies the detrimental effects of psychological distress (e.g., anxiety) on cognitive function. Specifically, the adverse impact of anxiety symptoms on cognition may be significantly amplified in individuals who are in a pre-frail state. This hypothesis integrates physical vulnerability with psychopathological processes, offering a novel perspective for elucidating the complex mechanisms of insomnia-related cognitive impairment. The present study therefore aimed to examine the interrelationships among insomnia, frailty, anxiety, and cognitive function in middle-aged and older adults, testing a moderated mediation model in which frailty moderates the anxiety-mediated pathway from insomnia to cognitive decline.

## 2 Methods

### 2.1 Study design and setting

This cross-sectional study was conducted at the First Affiliated Hospital of Anhui University of Chinese Medicine. It aimed to systematically investigate the interrelationships among frailty, anxiety symptoms, and cognitive function in middle-aged and older adults with insomnia, with a primary focus on testing the mediating roles of frailty and anxiety symptoms in the pathway between insomnia severity and cognitive performance. All data were collected during a single assessment session.

### 2.2 Participant recruitment and sample size

#### 2.2.1 Study population and recruitment

Participants were consecutively recruited from community settings and the outpatient and inpatient services of the Neurology, Geriatrics, and Cardiology departments at the First Affiliated Hospital of Anhui University of Chinese Medicine between August 2025 and February 2026. Research assistants initially screened potentially eligible individuals. A total of 200 individuals were assessed for eligibility. Of these, 59 did not meet the criteria for insomnia (PSQI global score ≤ 7), 18 were unable to complete the full assessment battery, and 31 declined to participate. A final sample of 108 participants was included in the study. Those who met the preliminary criteria received a comprehensive explanation of the study’s purpose, procedures, and their rights. Following initial verbal agreement, a rigorous eligibility review was conducted based on the following criteria:

#### 2.2.2 Inclusion criteria

(1) Aged 45 years or older;(2) Having a Pittsburgh Sleep Quality Index (PSQI) global score > 7; (3) Being conscious and able to complete the rater-administered assessments with standardized instructions. For self-reported measures, participants were required to possess basic reading or auditory comprehension skills, allowing for completion with assistance if needed;(4) Voluntarily providing written informed consent.

#### 2.2.3 Exclusion criteria

(1) Insomnia secondary to other severe somatic conditions (e.g., uncontrolled pain, severe respiratory failure) or primary psychiatric disorders (e.g., major depressive disorder, schizophrenia);(2) Diagnosed with major neurological diseases (e.g., dementia, Parkinson’s disease, significant sequelae of stroke);(3) Presence of severe visual or hearing impairment that would preclude participation in the assessments.

Participants who fulfilled all criteria and provided informed consent then received detailed, standardized instructions on the assessment procedures from uniformly trained research assistants.

Data for this cross-sectional study were collected in a single session comprising both self-reported and rater-administered instruments. The PSQI and the FRAIL Scale were completed as self-administered questionnaires, with a research assistant available to provide neutral reading assistance or verbatim transcription for participants needing help, ensuring no influence on responses. Concurrently, trained research assistants administered the following assessments in face-to-face interviews: the Montreal Cognitive Assessment—Basic (MoCA-B) for global cognition, and the Hamilton Anxiety Rating Scale (HAMA). All raters underwent standardized training to ensure consistent administration and scoring procedures.

### 2.3 Study instruments and measurements

#### 2.3.1 Assessment of insomnia severity

The Chinese version of the PSQI was used in this study to assess the severity of insomnia in patients. As an internationally recognized tool for evaluating sleep quality, the PSQI is well-suited for middle-aged and older adult populations. The index comprises 19 self-rated items, generating seven component scores: subjective sleep quality, sleep latency, sleep duration, habitual sleep efficiency, sleep disturbances, use of sleeping medication, and daytime dysfunction. The global PSQI score ranges from 0 to 21, with a total score > 7 adopted in this study as the cut-off point for insomnia. A higher score indicates poorer sleep quality and more severe insomnia. The Chinese version of the PSQI has demonstrated good reliability and validity in the Chinese population (22).

#### 2.3.2 Assessment of frailty status

The FRAIL scale was employed to assess the physical frailty status of participants. This scale consists of five items evaluating fatigue, resistance, ambulation, illnesses, and loss of weight. A response of “yes” to each item scores 1 point, yielding a total score ranging from 0 to 5. Based on their scores, participants were categorized into three levels: robust (0 points), pre-frail (1–2 points), and frail (3–5 points). Owing to its brevity and efficiency, the FRAIL scale has been widely used in numerous studies, including those involving Chinese older adults, and demonstrates good applicability (23).

#### 2.3.3 Assessment of cognitive function

The Beijing version of the MoCA-B was utilized to evaluate participants’ global cognitive function. The MoCA-B is a cognitive screening tool optimized for the Chinese population, covering eight cognitive domains: attention, executive functions, memory, language, visuospatial abilities, abstraction, calculation, and orientation. The total score is 30, and a score < 24 typically suggests cognitive impairment. This scale exhibits high sensitivity and specificity in identifying mild cognitive impairment and has been extensively validated in Chinese community and clinical populations (24).

#### 2.3.4 Assessment of anxiety symptoms

The severity of anxiety symptoms was assessed using the Chinese versions of the HAMA. The HAMA was administered by uniformly trained research assistants through clinical interviews assessing the patient’s condition over the past week. It contains 14 items encompassing anxious mood, tension, fears, insomnia, cognitive difficulties, depressed mood, somatic anxiety, and behavior at interview. Most items are rated on a 5-point scale from 0 to 4. A higher total score indicates more severe anxiety symptoms, with a total score ≥ 14 generally suggesting clinically significant anxiety symptoms. The HAMA is widely used in Chinese clinical settings with good reliability and validity, serving as one of the gold standards for assessing the severity of neurotic and depressive states.

### 2.4 Statistical analysis

Descriptive statistics were presented as mean ± standard deviation for continuous variables and number (percentage) for categorical variables. Group comparisons (e.g., by gender) were performed using independent samples t-tests or Mann-Whitney U tests for continuous variables, and chi-square or Fisher’s exact tests for categorical variables. A two-sided p-value < 0.05 was considered statistically significant.

Multiple linear regression analyses were employed to examine the associations between frailty, anxiety symptoms, and cognitive function. All models were adjusted for covariates including demographic factors (gender, age, years of education), insomnia characteristics (PSQI total score, duration of insomnia), and the number of comorbidities. Before modeling, multicollinearity was assessed using the variance inflation factor (VIF), with a threshold of VIF < 5 deemed acceptable. To mitigate the risk of Type I error inflation due to multiple comparisons (across global cognition and specific cognitive domains), a conservative significance threshold of p < 0.01 was applied.

The core analysis tested a moderated mediation model using the PROCESS macro for SPSS (version 4.0, Model 7). In this model, insomnia severity (PSQI total score) served as the independent variable (X), global cognitive function (MoCA−B total score) as the dependent variable (Y), anxiety (HAMA) symptoms as parallel mediators (M), and frailty status (coded as dummy variables with ‘Robust’ as the reference) as the moderator (W). Model 7 specifically assesses whether the moderator influences the strength of the path from insomnia to anxiety symptoms (the a−path, X to M). The significance of the interaction term (X×W) was first evaluated. If significant, the conditional indirect effects of insomnia on cognition at different levels of frailty (e.g., Robust vs. Pre−frail) were examined using bias−corrected bootstrap 95% confidence intervals based on 5, 000 resamples. A significant index of moderated mediation was used to confirm that the mediation effect is contingent on the level of the moderator.

Participants with missing data on any variable included in the analyses were excluded using listwise deletion. The randomness of missing data was confirmed using Little’s Missing Completely at Random (MCAR) test (p = 0.085). All analyses were conducted using SPSS 25.0 and the PROCESS macro.

### 2.5 Ethical considerations

All participants provided written informed consent prior to the study. The protocol was approved by the Ethics Committee of the First Affiliated Hospital of Anhui University of Chinese Medicine (Approval No. 2025AH-122) and was conducted in accordance with the ethical principles of the Declaration of Helsinki. To ensure confidentiality, participants were assigned code numbers, and all personal identifying information was kept confidential.

## 3 Results

### 3.1 Sample characteristics and descriptive statistics

The internal consistency of each scale was acceptable (Cronbach’s α: PSQI = 0.834, FRAIL = 0.712, HAMA = 0.899, **Supplementary Table 1**).

A total of 108 middle-aged and older adult patients with insomnia were included in this study. The mean age of participants was 59.62 ± 5.36 years; 41 (38.0%) were male and 67 (62.0%) were female. The average years of education was 12.05 ± 2.52 years. The mean PSQI score was 12.37 ± 4.17, indicating moderate insomnia severity. According to the FRAIL scale, the proportions of robust, pre-frail, and frail participants were 12.1%, 57.4%, and 30.5%, respectively. The mean HAMA score was 28.06 ± 12.64, exceeding the clinical threshold for significant anxiety symptoms. The mean MoCA-B score was 24.42 ± 1.99, suggesting mild cognitive impairment. No significant differences were observed between male and female participants on any demographic or clinical variables (all p > 0.05; **Table 1**).

**Table 1:** Baseline characteristics of the study population.

Characteristics	Total (n=108)	Male (n=41)	Female (n=67)	P value
Age (Mean ± SD)	59.62± 5.36	60.63 ± 4.43	59 ± 5.8	0.151
Years of Education (Mean ± SD)	12.05 ± 2.52	12.34 ± 2.64	11.87 ± 2.45	0.361
Disease Duration	5.51 ± 3.18	5.21 ± 3.17	5.76 ± 3.18	0.443
Hypertension	52	23	29	0.326
Type 2 diabetes	50	20	30	0.843
PSQI Score	12.37 ± 4.17	12.51 ± 4.32	12.28 ± 4.1	0.814
MoCA-B Score	24.42 ± 1.93	24.48 ± 2.05	24.37 ± 1.97	0.822
FRAIL Score	2.12 ± 1.69	2.26 ± 1.8	2.03 ± 1.6	0.606
HAMA Score	28.06 ± 13.05	28.02 ± 13.61	28.19± 13.34	0.66

### 3.2 Associations among frailty, anxiety, insomnia severity, and cognitive function

Using robust as the reference, both pre-frailty and frailty significantly predicted lower cognitive levels (pre-frail: β = -2.362, 95% CI [-3.102, -1.322], p < 0.001; frail: β = -3.513, 95% CI [-4.629, -2.396], p < 0.001). Pre-frailty also significantly predicted higher levels of anxiety symptoms (β = 13.128, 95% CI [5.91, 20.346], p < 0.001), while frailty did not (β = 7.238, 95% CI [-0.511, 14.986], p = 0.067). Both pre-frail and frail individuals had significantly higher PSQI scores compared to robust individuals (pre-frail: β = 2.496, 95% CI [0.017, 4.975], p = 0.048; frail: β = 3.319, 95% CI [0.658, 5.981], p = 0.015; **Table 2**).

**Table 2:** Relationships of anxiety, insomnia, and cognition with different frailty levels .

Characteristics	Frailty Level	β	P Value	95%Cl	
				Lower limit	Upper limit
HAMA	Robust​	–	–	–	–
	Pre-frailty	13.128	0.000	5.91	20.346
	Frailty	7.238	0.067	-0511	14.986
Cognition	Robust​	–	–	–	–
	Pre-frailty	-2.362	0.000	-3.102	-1.322
	Frailty	-3.513	0.000	-4.629	-2.396
PSQI	Robust​	–			
	Pre-frailty	2.496	0.048	0.017	4.975
	Frailty	3.319	0.015	0.658	5.981

#### 3.2.2 Relationship between cognition and anxiety symptoms, frailty status, and insomnia

A multiple linear regression analysis was conducted with MoCA-B total score as the dependent variable, controlling for demographic variables including age, sex, and years of education. The results showed that frailty (β = −1.3, 95% CI [−1.737, −0.862], p < 0.001), insomnia severity (β = −0.176, 95% CI [−0.261, −0.091], p < 0.001), and anxiety symptoms (β = −0.036, 95% CI [−0.064, −0.009], p = 0.010) were each significantly associated with lower cognitive function. Age (β = −0.021, p = 0.404), years of education (β = −0.038, p = 0.478), and sex (β = −0.278, p = 0.323) were not significant predictors (**Table 3**). Using a conservative significance threshold of p < 0.01, the effects of frailty and insomnia severity remained significant.

**Table 3:** Relationship between cognition and anxiety symptoms, frailty status, and insomnia.

Characteristics	β	P value	95%Cl	
			Lower limit	Upper limit
HAMA	-0.036	0.010	-0.064	-0.009
PSQI	-0.176	0.000	-0.261	-0.091
Frailty	-1.3	0.000	-1.737	-0.862
Age	-0.021	0.404	-0.072	0.029
Years of Education	-0.038	0.478	-0.144	0.068
Sex	-0.278	0.323	-0.833	0.277

### 3.3 Simple mediation analysis: anxiety as a mediator

To examine whether anxiety symptoms mediated the relationship between insomnia severity and cognitive function, a simple mediation analysis was performed using PROCESS Model 4 with 5, 000 bootstrap resamples. The total effect of insomnia on cognition was significant (β = -0.285). After accounting for anxiety symptoms, the direct effect of insomnia on cognition remained significant (β = -0.233, 95% CI [-0.327, -0.138], p < 0.001). However, the indirect effect through anxiety was not statistically significant (β = -0.052, Bootstrap 95% CI [-0.127, 0.008]), indicating that anxiety alone did not mediate the insomnia-cognition relationship (**Table 4**).

**Table 4:** Results of the mediation analysis with anxiety symptoms as the mediator.

Path analysis	β	Bootstrap SE	95% CI	P
Total Effect	-0.285	–	–	–
Direct Effect	-0.233	0.047	-0.327, -0.138	0.000
Indirect Effect	-0.052	0.034	-0.127, 0.008	–

### 3.4 Moderated mediation analysis: frailty as a moderator

Given the non-significant simple mediation, we next tested whether pre-frailty moderates the indirect pathway. A moderated mediation analysis was conducted using PROCESS Model 7, with pre-frailty (0 = robust, 1 = pre-frail) as the moderator of the insomnia–anxiety path (a-path).

The interaction between insomnia severity and pre-frailty was significant in predicting anxiety symptoms (PSQI × Pre-frailty: β = 1.0294, 95% CI [0.1864, 1.8725], p = 0.0172; **Table 5**), (PSQI × Frailty: β = −1.213, 95% CI [−2.176, −0.250], p = 0.014; **Table 6**), indicating that pre-frailty significantly moderated the insomnia–anxiety relationship. Simple slope analyses revealed that insomnia severity positively predicted anxiety symptoms in both groups, with a stronger effect in the pre-frail group (β = 2.4952, 95% CI [1.9136, 3.0768], p < 0.001) than in the robust group (β = 1.4658, 95% CI [0.8555, 2.0760], p < 0.001).

**Table 5:** Moderating effect of pre-frailty on the insomnia–anxiety relationship.

Variable	β	P-value	Bootstrap 95% CI
PSQI (X)	1.465	0.000	0.855, 2.076
Pre-frailty (W)	−3.825	0.494	−14.893, 7.243
PSQI ×Pre-frailty	1.029	0.017	0.186, 1.872

**Table 6:** Moderating effect of frailty on the insomnia–anxiety relationship.

Variable	β	P-value	Bootstrap 95% CI
PSQI (X)	2.518	0.000	1.958, 3.079
Frailty (W)	10.111	0.128	−2.985, 23.208
PSQI × Frailty	−1.213	0.014	−2.176, −0.250

The conditional indirect effects of insomnia on cognition through anxiety were significant in both groups (**Table 7**). The indirect effect was significant in the robust group (β = −0.0527, Bootstrap 95% CI [−0.1108, −0.0045]) and was significantly stronger in the pre-frail group (β = −0.0897, Bootstrap 95% CI [−0.1787, −0.0068]). The index of moderated mediation was significant (Index = −0.0370, Bootstrap 95% CI [−0.1007, −0.0003]), confirming that the anxiety-mediated indirect effect of insomnia on cognition differed significantly between robust and pre-frail individuals, with the pathway being more pronounced in the pre-frail state.

**Table 7:** Moderated mediation analysis: the role of frailty in the insomnia-anxiety-cognition pathway.

Moderator level	Indirect effect	Bootstrap SE	95% CI
Robust	−0.052	0.027	−0.110, −0.004
Pre-frailty	−0.089	0.043	−0.178, −0.006
Index of Moderated Mediation	−0.037	0.026	−0.100, −0.0003

The b-path (HAMA → cognition) was significant (β = −0.0360, p = 0.0158), indicating that higher anxiety was associated with lower cognitive function. After controlling for anxiety and covariates, the direct effect of insomnia severity on cognitive function remained significant (β = −0.2116, 95% CI [−0.3042, −0.1190], p < 0.001).

## 4 Discussion

To our knowledge, this study is among the first to systematically elucidate the complex relationships among anxiety symptoms, frailty, and cognitive function in middle-aged and older adults with insomnia using a moderated mediation model. The key finding of this study is that pre-frailty significantly moderates the anxiety-mediated pathway from insomnia to cognitive decline. The conditional indirect effect of insomnia on cognition through anxiety was significant in both robust and pre-frail individuals, with the index of moderated mediation confirming that the indirect effect was significantly stronger in the pre-frail group. This demonstrates that while the anxiety-mediated mechanism is active across both states, pre-frailty amplifies this psychological pathway, identifying it as a distinct vulnerable window in which anxiety plays a particularly pronounced role in linking insomnia to cognitive decline. This significant moderated mediation effect provides a critical explanation for the inconsistent findings in previous research regarding the anxiety symptoms-cognition relationship (18, 25). Specifically, the negative indirect pathway from insomnia to anxiety symptoms to cognitive decline​ was significant significantly amplified among individuals in the pre-frail state.

Our study found that compared to their robust counterparts, individuals with insomnia who were pre-frail exhibited a higher propensity for anxiety symptoms, and cognitive decline. The moderating effect of frailty on the relationship between insomnia and anxiety symptoms can be interpreted through multiple physiological and psychosocial mechanisms. From a physiological perspective, the reserve capacity theory offers a robust framework (26). Individuals with frailty experience a significant decline in physiological and psychological reserves, diminishing their capacity to cope with chronic stressors like insomnia. When confronted with sleep disturbances, the hypothalamic-pituitary-adrenal (HPA) axis in frail individuals is more susceptible to hyperactivation (27), leading to disrupted cortisol secretion rhythms and elevated levels of pro-inflammatory cytokines (e.g., IL-6, TNF-α). This creates a vicious cycle within the neuroendocrine-immune system, potentially triggering or exacerbating anxiety symptoms (28, 29). The depletion of physiological reserves heightens the sensitivity of pre-frail individuals to insomnia, thereby precipitating negative emotions such as anxiety. From a psychosocial standpoint, the biopsychosocial model provides another lens, positing that health outcomes are determined by complex interactions among biological, psychological, and social factors (30). Frailty itself constitutes a chronic stressor. When compounded with insomnia, the combined burden may exceed an individual’s psychological threshold, leading to a significant increase in anxiety symptoms (31, 32). Our results align well with this model. We found that frailty significantly moderated the effect of insomnia on anxiety symptoms. Simple slope analysis further confirmed that the predictive power of insomnia on anxiety symptoms strengthened with increasing frailty levels. Frail and older individuals exhibit diminished physical and psychological reserves, making them more vulnerable to external stressors (33), including age-related pressures and cumulative exposures such as COVID-19 infection (34). Inability to cope with these challenges may undermine self-confidence, which in turn could increase psychological burden, leading to anxiety or even depression. Meanwhile, this positive correlation between cognitive decline and frailty has been consistently demonstrated, including among immigrants (35). In depressed populations, there are direct and indirect links between frailty and depression, mediated by anger, resilience, and anxiety (36). A large prospective cohort study found that frailty significantly influenced the transition from baseline status to single mental disorders, their comorbidity, and mortality (37). Furthermore, frailty may impair social functioning in individuals with insomnia, reducing their contact with social support networks. Since social support is negatively correlated with insomnia, anxiety, and depression (38), and as insomnia partially mediates the relationship between social support and negative emotions, the weakening of these protective factors can psychologically weaken elderly insomnia patients. Therefore, frailty exacerbates anxiety and depression (39, 40) and reduces self-management capacity for coping with disease symptoms and daily demands, which may be one reason for cognitive decline.

One unexpected finding of this study was the differential pattern observed across the two frailty models. In the pre-frailty model, the anxiety-mediated indirect effect was significant in both groups and was significantly amplified in pre-frail individuals. In the frailty model, although the conditional indirect effects were also significant in both robust and frail groups, the index of moderated mediation did not reach significance. This contrast between the two models further reinforces that pre-frailty represents a critical transition state where psychological pathways are particularly modifiable. As individuals progress to full frailty, direct biological mechanisms—including chronic low-grade inflammation, persistent HPA axis dysregulation, and accumulated vascular or metabolic brain damage—may increasingly dominate the pathway to cognitive decline, partially supplanting the anxiety-mediated route. The direct effect of frailty on cognition was very strong, supporting this interpretation. Future studies with balanced sample sizes across frailty levels are needed to further test this proposed non-linear pattern.

Our findings help resolve previous inconsistencies in the literature regarding the anxiety-cognition relationship. Studies that did not account for frailty as a moderator may have averaged effects across robust, pre-frail, and frail individuals, thereby obscuring the differential strength of the anxiety-mediated pathway across frailty states. The most important implication of our study is that it offers a reasonable explanation for the inconsistent findings in previous research on the anxiety-cognition relationship. Many studies, potentially by failing to consider frailty as a key moderator, may have masked or diluted simple mediation effects. Our results indicate that frailty defines a specific psychophysiological vulnerable subgroup in which the pathway from insomnia to cognition via anxiety is significantly activated. This highlights the importance of including moderating variables when examining complex psychopathological processes. Regarding clinical relevance, a prospective study showed that frailty was significantly associated with depression, anxiety, and their comorbidity, and frailty (37), especially in older women, was associated with poorer cognition (41), and the co-occurrence of frailty and cognitive decline is associated with impaired self-determination​ (42). A meta-analysis found that baseline frailty was significantly associated with an increased risk of cognitive impairment in the elderly (p = 0.02), with frail older adults being more prone to cognitive impairment (43). Our study categorized individuals into robust, pre-frail, and frail. This finer classification across the frailty spectrum may have contributed to observing significant associations in our findings. Unlike meta-analyses that dichotomize participants into frail and non-frail, the early and pre-frail stages represent a critical window for interventions that might improve patients’ overall insomnia scores and anxiety symptoms.

Another important finding is that the direct effect of insomnia on cognitive function remained significant after controlling for anxiety symptoms. This aligns with existing evidence that insomnia may impair cognition through direct pathways such as disrupting sleep-dependent memory consolidation processes (44–46) and affecting the clearance efficiency of metabolic waste from the brain during sleep (47). Simultaneously, under a conservative significance level, both frailty and insomnia severity remained independent significant predictors of cognitive function, further underscoring their important roles in the cognitive health of this population.

It is important to acknowledge that frailty shares conceptual overlap with fatigue, reduced activity, and psychological distress, which may influence anxiety and cognitive performance. Nevertheless, the moderated mediation model revealed a significant conditional indirect effect, suggesting that frailty plays a moderating—not merely overlapping—role in the insomnia-anxiety-cognition pathway.

In conclusion, pre-frailty significantly moderates the insomnia–anxiety–cognitive performance pathway, with the anxiety-mediated indirect effect being significantly amplified in pre-frail individuals compared to their robust counterparts. This finding identifies pre-frailty as a distinct psychologically vulnerable state in which the anxiety pathway from insomnia to cognitive decline is particularly pronounced and potentially modifiable. Clinicians should assess frailty status in middle-aged and older adults with insomnia, as pre-frail individuals may represent a critical window for integrated interventions targeting anxiety to preserve cognitive health.

## 5 Limitations

Several limitations of the present study warrant acknowledgment. First, the cross-sectional design precludes causal inference regarding the temporal sequence or directionality of the observed associations. Longitudinal investigations are necessary to elucidate the dynamic interrelationships among insomnia, frailty, anxiety symptoms, and cognitive decline over time. Second, the sample was recruited exclusively from a single tertiary hospital, which may limit the generalizability of the findings to community-dwelling or more heterogeneous populations. Third, all assessments relied on subjective self-report and rater-administered instruments. The incorporation of objective measures—such as actigraphy, polysomnography, or neuroimaging biomarkers—would substantially strengthen the validity and reproducibility of future studies.

Fourth, this study did not differentiate among clinically distinct insomnia subtypes, including sleep-onset insomnia, sleep-maintenance insomnia, and early-morning awakening. Given the pathophysiological heterogeneity of insomnia, different phenotypes may exhibit divergent associations with affective symptoms and cognitive outcomes. Consequently, it remains uncertain whether the moderated mediation model identified herein applies uniformly across specific insomnia presentations. Future studies with larger, phenotypically well-characterized samples are needed to address this question.

Fifth, the present investigation examined dimensional symptom severity on the HAMA, and MoCA-B rather than formally diagnosed clinical syndromes. Accordingly, the findings pertain to the burden of anxiety symptoms, depressive symptoms, and cognitive symptoms in the context of insomnia and frailty, rather than to established psychiatric or neurocognitive disorders. Moreover, cognitive performance was assessed globally using a brief screening tool, which precludes inferences regarding domain-specific impairments (e.g., in memory, executive function, or attentional control). Future studies should employ comprehensive neuropsychological batteries to determine whether the observed effects are specific to certain cognitive domains.

Sixth, the HAMA includes items that assess insomnia symptoms (e.g., difficulty falling asleep, restless sleep), which may partially overlap with the PSQI measure of insomnia severity. This potential item overlap could inflate the observed association between insomnia and anxiety and should be considered when interpreting the insomnia–anxiety relationship. Future studies may benefit from using anxiety measures that do not include sleep-related items, or from employing statistical corrections for item overlap.

## Data Availability

The raw data supporting the conclusions of this article will be made available by the authors, without undue reservation.
